# Long-term High Fat Ketogenic Diet Promotes Renal Tumor Growth in a Rat Model of Tuberous Sclerosis

**DOI:** 10.1038/srep21807

**Published:** 2016-02-19

**Authors:** Arkadiusz D. Liśkiewicz, Daniela Kasprowska, Anna Wojakowska, Krzysztof Polański, Joanna Lewin–Kowalik, Katarzyna Kotulska, Halina Jędrzejowska–Szypułka

**Affiliations:** 1 Medical University of Silesia, School of Medicine in Katowice, Department of Physiology, Katowice, Poland; 2 Affective Cognitive Neuroscience Lab, Department of Behavioral Neuroscience and Drug Development, Institute of Pharmacology Polish Academy of Sciences Krakow, Poland; 3 Medical University of Silesia, School of Medicine in Katowice, Department of Pharmacology, Katowice, Poland; 4 Laboratory of Molecular Biology, Faculty of Physiotherapy, The Jerzy Kukuczka Academy of Physical Education, Katowice, Poland; 5 Maria Sklodowska-Curie Memorial Cancer Center and Institute of Oncology, Gliwice Branch, Poland; 6 Warwick Systems Biology Centre, University of Warwick, CV4 7AL, UK; 7 Department of Neurology and Epileptology, The Children's Memorial Health Institute, Warsaw, Poland

## Abstract

Nutritional imbalance underlies many disease processes but can be very beneficial in certain cases; for instance, the antiepileptic action of a high fat and low carbohydrate ketogenic diet. Besides this therapeutic feature it is not clear how this abundant fat supply may affect homeostasis, leading to side effects. A ketogenic diet is used as anti-seizure therapy i.a. in tuberous sclerosis patients, but its impact on concomitant tumor growth is not known. To examine this we have evaluated the growth of renal lesions in Eker rats (*Tsc2+/−*) subjected to a ketogenic diet for 4, 6 and 8 months. In spite of existing opinions about the anticancer actions of a ketogenic diet, we have shown that this anti-seizure therapy, especially in its long term usage, leads to excessive tumor growth. Prolonged feeding of a ketogenic diet promotes the growth of renal tumors by recruiting ERK1/2 and mTOR which are associated with the accumulation of oleic acid and the overproduction of growth hormone. Simultaneously, we observed that Nrf2, p53 and 8-oxoguanine glycosylase α dependent antitumor mechanisms were launched by the ketogenic diet. However, the pro-cancerous mechanisms finally took the ascendency by boosting tumor growth.

Tuberous Sclerosis Complex (TSC) is a multi-system genetic disease caused by autosomal dominant mutations in the tumor suppressor genes encoding hamartin (*TSC1*) or tuberin (*TSC2*). Mutations in these genes result in the development of mostly non-neoplastic lesions which are found in many organs^1^. These benign tumors can eventually develop into neoplastic ones. For example, a subependymal giant cell astrocytoma develops from hamartomous subependymal nodules^2^. Neoplastic lesions in TSC can also be found in the lungs and kidneys^3^. Renal neoplasms such as angiomyolipoma or renal cell carcinoma exhibit invasive and/or aggressive phenotypes^4^,^5^,^6^. The first one (invasive) is typical for renal cell carcinoma^7^, while the latter (aggressive) is observed particularly in large angiomyolipomas and manifests as hemorrhages which are the main cause of death in TSC patients^8^. Additionally, brain lesions in TSC are often associated with severe symptoms: hydrocephalus and epilepsy^9^,^10^. The seizures observed in the course of TSC are mostly resistant to standard medical treatment; thus, a high fat, low carbohydrate ketogenic diet (HFKD) is often applied as an add-on therapy^11^. HFKD is widely used as a treatment in epilepsy and other seizure-related disorders, where some patients require long-term dietary management to achieve therapeutic efficiency^12^. Unfortunately, little is known about the influence of HFKD on tumorigenesis, and the influence of such a diet on the development of TSC-related lesions has not yet been studied.

Nourishment provides many exogenous factors which may affect the mechanisms underpinning cancerogenesis. Despite the growing knowledge about cancer biology and treatment, it is still not clear how different aspects of nutrition are implicated in the progression of the disease. Diets, like many other factors, can be beneficial but can also lead to some unintended side effects. In terms of current knowledge, nutritional balance seems to be the most reasonable approach. Ketogenic diets are associated with an asymmetric supply of fats or proteins, in relation to carbohydrates. It is believed that HFKD is a therapeutic option for cancer and is able to support conventional treatments such as chemotherapy and radiotherapy, particularly in brain tumors^13^. The distribution of its ingredients with sugar limitations can be disadvantageous for hyperproliferative cells and can lead to tumor regression^14^. However, it should be taken under consideration that a long-term abundant supply of lipids may have an additional impact on tumor growth^15^, and especially high fat diets can also promote tumorigenesis, as reported previously^16^,^17^.

Based on the aforementioned, we believed that there was a need to evaluate how long-term HFKD might affect the development of TSC renal tumors. For this purpose, we used Eker rats with a spontaneous mutation in the *Tsc2* gene, which are an excellent animal model for studying TSC-related tumors^18^. In Eker rats, the tendency to develop renal tumors increases with age and they are found in nearly all 1-year-old animals^19^. These tumors are classified as adenocarcinoma and renal cell carcinoma; thus, Eker rats are also useful in renal carcinoma research^20^,^21^. In our project, Eker rats were fed with modified HFKD for four, six and eight months. Morphometric data was supported with a biochemical analysis to reveal the molecular mechanisms of HFKD action on renal tumorigenesis.

## Materials and Methods

### Ketogenic diet

The HFKD was prepared by Morawski (Kcynia, Poland) from lard, butter, corn oil, casein, wheat bran, a mineral mix, a vitamin mix and dextrose. The nutritional profile of the diet was: fat 79%, protein 9.5%, carbohydrates 0.8%, fiber 5%, vitamins and minerals 5.7%. The diet was prepared according to the BioServ F3666 Ketogenic Diet (Frenchtown, NJ, USA) recipe, and was modified by the replacement of cellulose with wheat bran. This modification was introduced due to the results of the preliminary study (see below in Results section). The standard fodder was taken from the same supplier (see also Supplementary Information).

### Animals and project design

All animals were provided by the Animal House of the Experimental Medicine Center, Medical University of Silesia, Katowice, Poland and were treated in accordance to the Directive 2010/63/EU for animal experiments using the protocols approved and monitored by the Local Committee for Animal Experiments of the Medical University of Silesia.

The Eker rat (Long Evans *Tsc2+/−*) husbandry was derived from Robert Waltereit, University of Heidelberg, Mannheim, Germany. The animals were inbred and their genotypes were determined by PCR^22^. As a result, 93 adult (52 males and 41 females) Eker rats were divided into three ketogenic and one control group: [1] KD4, where 16 animals (9 males and 7 females) were treated with HFKD from 10 mo. of age for the next four months; [2] KD6, where 26 animals (15 males and 11 females) were treated with HFKD from 8 mo. of age for the next six months; [3] KD8, where 17 animals (9 males and 8 females) were treated with HFKD from 6 mo. of age for the next eight months; and [4] ST, the control group, where 34 animals (19 males and 15 females) were housed on the standard rodent fodder. Additionally, six wild-type Long Evans rats were used for protein measurements conducted by Western Blotting. Three of them were maintained on the standard diet (LE ST) and the remaining on an HFKD similarly to the KD6 group (LE KD). All the animals were anesthetized (i.p. injection of 100 mg/kg ketamine plus 10 mg/kg xylazine) and sacrificed at the age of 14 mo. by a transcardiac perfusion with 200 mL of Tris-Buffered Saline (TBS) (pH 7.4, 4 °C) followed by 200 mL of 10% formalin in TB (pH 7.4, 4 °C). Immediately after the TBS and before the fixative perfusion, appr. 100 mg of normal kidney tissue (cortex) was collected and snap-frozen in liquid nitrogen, then stored at −80 °C. This renal samples were used in Western blot and metabolome analysis.

### Tumor assessment

For the evaluation of the renal tumor size, visible solid tumors with a diameter >2 mm were measured with a digital caliper. The length and width of the lesion were measured and calculated by the formula: tumor volume = 3.14/6 × a^2^ × b, where “a” is the shorter and “b” is the longer axis of the tumor^23^. The gross tumor volume has been expressed as a mean of the tumor volume per group and a sum of all tumor volumes per animal. To confirm the data obtained from the macroscopic evaluation, sets of 2 mm interval sections from KD6 and ST groups (Rat Kidney Slicers, Zivic Instruments, Pittsburgh, PA, USA) were prepared as H&E-stained paraffin 5 micrometer sections. These sections were photographed (63× magnification, Wild M400, ProMicron, Kirchheim, Germany) to obtain high-resolution images (5184 × 3456 pixels) and each tumor or cyst identified was measured using ImageJ (NIH, Bethesda, MD, USA) to determine its length and width, as well as the percent of the lumen filled by tumor (this was 0% for a simple cyst, and 100% for a completely filled, solid adenocarcinoma). These measurements were converted into the tumor volume per lesion using the following formula: Tumor volume = maximum(tumor percent, 5)/100 * 3.14159/6 * 1.64 * (tumor length * tumor width)^1.5. The total tumor volume per animal was then equal to the sum of the tumor volumes of each lesion identified^24^. A microscopic evaluation was not conducted in one rat from the KD6 group, where the kidneys were extremely overgrown by tumor.

### Blood serum analyses

On the day of euthanasia, 1 mL of orbital sinus blood was collected 2 h after the lights were turned on, because growth hormone peaks are expected to occur especially during the first hours of the inactive period^25^. The blood was allowed to clot by leaving it for 30 min at room temperature. The samples were then centrifuged (2,000× g for 15 minutes), and the serum was collected and stored at −80 °C. The serum level of β-hydroxybutyrate was measured using a β-Hydroxybutyrate Assay Kit (BioVision, Milpitas, CA, USA). The serum levels of the insulin (Rat Insulin ELISA, Demeditec diagnostics GmbH, Germany), the insulin-like growth factor 1 (IGF-1; Quantikine ELISA Mouse/Rat IGF-1, R&D System, Minneapolis, MN, USA) and the growth hormone (Rat/Mouse GH, Millipore, St. Charles, MO, USA) were measured according to the manufacturer’s instructions. These measurements has not been performed in animals harboring pituitary tumors (23% in ST, 19% in KD4, 8% in KD6 and 28% in KD8 groups). The levels of glucose and triglycerides were measured with a glucometer (CardioCheck Professional, Indiana, IN, USA) in whole blood collected from the tail vein.

### Immunohistochemistry

Five micrometer paraffin sections were deparaffinized with a xylene and alcohol series, treated with Target Retrieval Solution pH 6.1 (Dako, Carpinteria, CA, USA), blocked with 3% H_2_O_2_ in methanol, and then placed in a 5% normal goat serum in 0.1% Triton X in TBS. The sections were incubated overnight with primary antibodies at 4 °C, were washed, and then incubated (2h) with a secondary antibody conjugated with horseradish peroxidase (HRP). AEC (3-amino-9-ethylcarbazole, Envision + System Dako) was then applied to generate a color reaction. The slides were then counterstained with hematoxylin. The primary antibodies used for labeling were: p-ERK1/2 Thr202/Thr204 (#9101, 1:100, Cell Signaling Technology); and p-mTOR Ser2448 (#2971, 1:100, Cell Signaling Technology). A visual qualitative observation (using BX51 microscope with U-TV-1X-2 camera and CellˆB image acquisition software, all from Olympus, Tokyo, Japan) was used to determine the signal intensity in the tumor tissue relative to the normal tissue.

### Immunoblotting

The protein level in the renal tissue was estimated by means of Western Blotting. Basing on the immunohistochemistry results, we analyzed the presence of p-ERK1/2 (Thr202/Thr204; #9101, 1:1000, Cell Signaling Technology) and p-mTOR (Ser2448; #2971, 1:1000, Cell Signaling Technology) to verify the activity of these proteins in the kidneys. To reveal other possible mechanisms implicated in the HFKD action, we also examined the amounts of p53 (MAB1355, 1:500, R&D), nuclear factor erythroid 2-related factor 2 (Nrf2; MAB3925, 1:1000, R&D) and 8-oxoguanine glycosylase α (NB100-106, 1:1000, Novus Biological). The results were normalized to the β-actin reference protein (#4970, 1:1000, Cell Signaling Technology) (see also Supplementary Information).

### Metabolite profiling

To complete the comprehensive analysis of the HFKD actions, we performed gas chromatography/mass spectrometry (GC/MS) – based profiling of metabolites present in rat kidney tissue extracts. Each tissue sample was processed and analyzed separately in at least three biological repetition for each animal group. Freshly frozen renal samples were extracted with mixtures of water and methanol, then methylene chloride and methanol as described previously^26^. Samples were subjected to GC/MS analysis directly after derivatization according to established protocols^26^ (please see also Supplementary information for more details). The obtained metabolomic data was analyzed to evaluate an influence of long-term feeding by HFKD on the renal metabolome changes.

### Statistical analysis

Statistica 10 (StatSoft, Inc., Tulsa, OK, USA) was used for the statistical analysis. Macroscopic tumor sizes and the serum levels of triglycerides, insulin and IGF-1 were tested using a non-parametric Kruskal-Wallis test. For comparison of the microscopic tumor volumes between the two analyzed groups (KD6 and ST) the Wilcoxon Mann-Whitney test was applied. Non-parametric data was presented as medians and 5–95 percentiles (enclosed in square brackets). All other data with a normal distribution was analyzed with ANOVA and presented as mean ± S.E.M.

In the metabolomic profiling, the data was filtered to only feature metabolites that showed up in at least 100% of the samples in each of the groups. The data was log-transformed, the normality was assessed with the Lilliefors composite goodness-of-fit test for each group, and homoscedasticity was assessed with Bartlett’s test. The data for each individual metabolite was analyzed with both ANOVA and a Kruskal-Wallis one-way analysis of variance. The P-values for each group’s normality testing, homoscedasticity testing, ANOVA and Kruskal-Wallis testing had the Benjamini-Hochberg FDR correction applied. After the FDR correction, the final test was chosen after assessing the normality and homoscedasticity of each metabolite’s data across the groups: if at least one of the groups was identified as not normal, or the Bartlett’s test P-value was significant, the Kruskal-Wallis was chosen as the test for the metabolite; otherwise, ANOVA was used as the test for the metabolite.

If the final chosen test P-value was significant (with a significance threshold of 0.05), post-hoc testing was also performed to identify between which groups the values were significantly different. In the case of ANOVA, the Fisher (Western Blotting data), Dunnett’s (ELISA’s parametric data) or Tukey test were used. In the case of Kruskal-Wallis, the Nemenyi test with the Dunn correction was used, and the test statistic was compared to the tabulated values for a significance threshold of 0.05. Figures were generated with Prism 5.01 (GraphPad Software, San Diego, CA, USA).

## Results

### The ketogenic properties of modified HFKD

**Figure 1:**
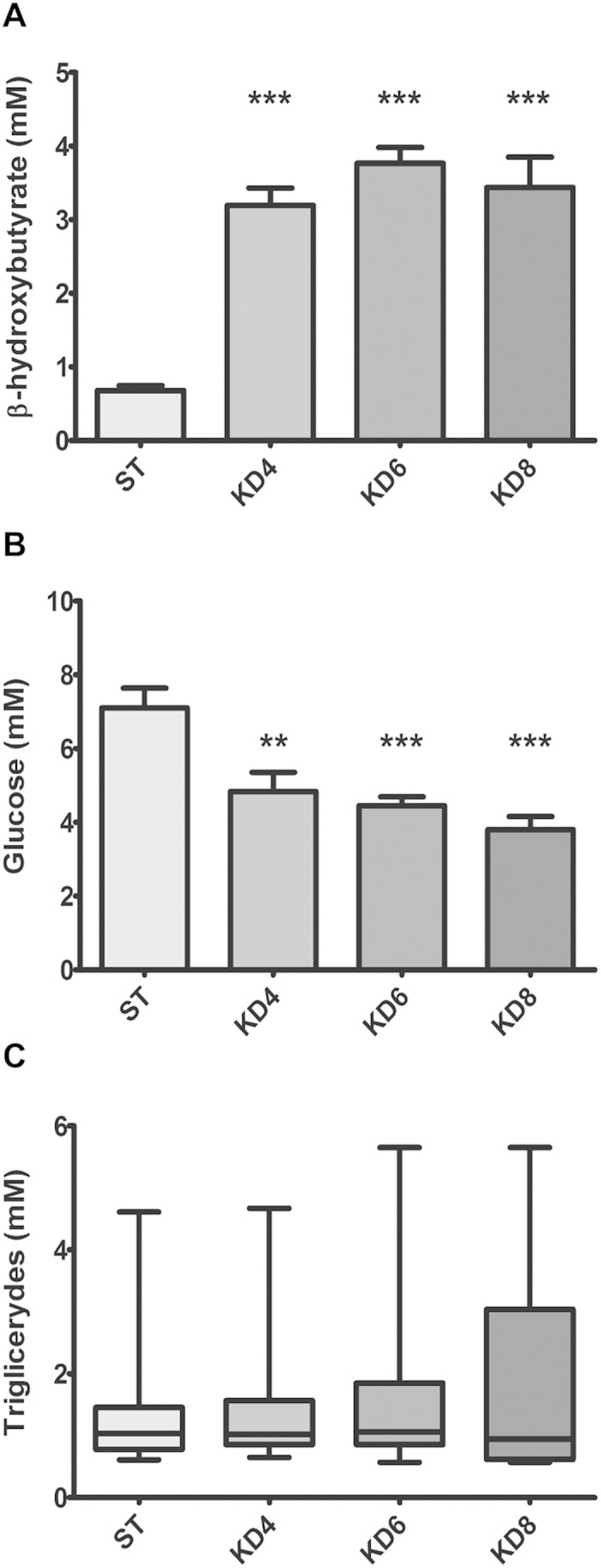
Measurements of blood substrate levels in Eker rats (Tsc2*+/−*). HFKD increases ketone body levels (**A**) with a decline in glucose concentrations (**B**) and unchanged triglyceride amounts (**C**). Results are given as a mean ±S.E.M ((**A**,**B**) ANOVA with a Tukey’s post hoc test) or median (**C**) whiskers represent 5–95 percentiles; Kruskal-Wallis ANOVA with a Dunn’s post hoc test). KD4, KD6, KD8 – animal groups treated with HFKD for four, six or eight mo., respectively; ST – control animals fed with a standard diet. **P < 0.01, ***p < 0.001 as compared to ST.

Considering the fact that prolonged nourishment on HFKD may influence tumor development, we aimed to perform a long-term study. During preliminary experiments, we observed total growth inhibition and emaciation in rats being fed with the original HFKD for longer than 2 months. Besides these strong side-effects, a high mortality rate was noted after 4 months of treatment (data not shown). To overcome this issue, we elaborated a modification of classical HFKD. The debilitating effects were not observed after the replacement of cellulose with wheat bran. Blood ketones and glucose levels were examined in order to demonstrate the ketosis induced by the modified HFKD. The obtained results showed a rise in the mean β-hydroxybutyrate plasma level (ST, 0.68 ± 0.1 mM; KD4, 3.2 ± 0.2 mM, KD6, 3.8 ± 0.2 mM; KD8, 3.4 ± 0.4 mM, Fig. 1A) with a concomitant decline in the mean glucose concentration (ST, 7.1 ± 0.5 mM; KD4, 4.8 ± 0.5 mM; KD6, 4.5 ± 0.2 mM; KD8, 4 ± 0.4 mM; Fig. 1B), which confirmed the ketogenic properties of the applied diet. Although there was an abundant fat supply, the median blood triglycerides were unchanged between the groups (ST, 1 mM [0.6–4.8]; KD4, 1mM [0.7–4.7]; KD6 1.1 mM [0.6–5.4]; KD8, 1 mM [0.6–5.7]; Fig. 1C).

### Long-term treatment with HFKD increases renal tumor growth in Eker rats

**Figure 2:**
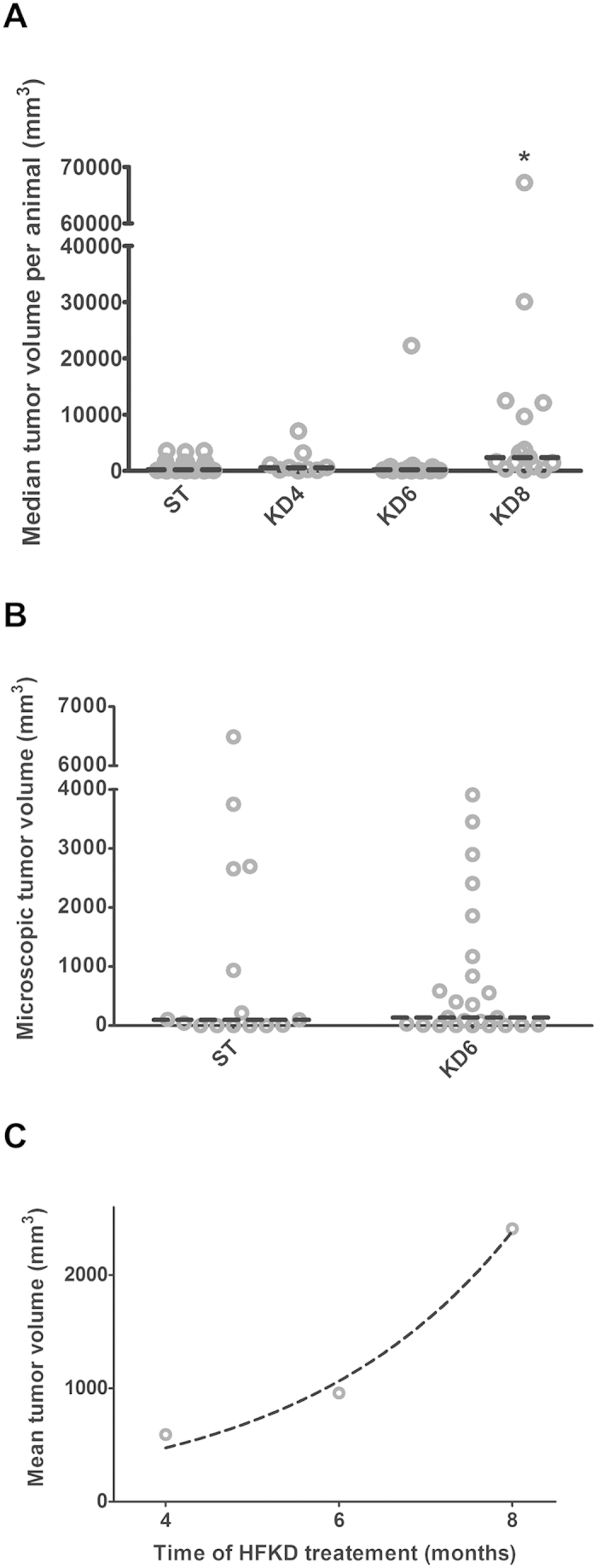
Macroscopic and microscopic renal tumor volumes in Eker rats (Tsc2**+/−**) treated with a ketogenic or a standard diet. (**A**) Macroscopic measurements of renal tumor volumes per single animal (*p < 0.05 as compared to ST for Kruskal-Wallis ANOVA with Dunn’s post hoc test). (**B**) Microscopic evaluation of renal tumor volumes per single animal (Wilcoxon Mann-Whitney test). (**C**) Exponential changes of mean tumor size per group in rats with the time of the HFKD treatment (R^2^ = 0.97). The dashed, horizontal line indicates the median tumor volume. KD4, KD6, KD8 – groups treated with HFKD for four, six or eight mo., respectively; ST – control animals fed with a standard diet.

At the age of 10, 8 and 6 mo. a rat standard fodder was replaced with HFKD – for 4, 6 and 8 months, respectively. We did not observe any influence of HFKD on tumor size between the KD4, KD6 and ST groups, where the median tumor volume was 568 [5–7084], 206 [5–22268] and 188 [7–3572] mm^3^, respectively (Fig. 2A). However, sustained treatment with HFKD led to an excessive growth of tumors in the KD8 group when compared to the control group (p = 0.018), with a median volume of 2377 [148–67256] mm^3^ (Fig. 2A). The incidence of tumors with a diameter >2 mm was higher in the groups where the rats were fed with HFKD (53% in KD4, 65% in KD6 and 84% in KD8) when compared with the control animals (48%). The results of a microscopic analysis of all lesions confirmed a lack of a differences between ST and KD6 groups with a median total tumor volume of 99 [0.01–6488] and 137 [1–3795] mm^3^, respectively (p = 0.55, Fig. 2B). The incidence of all renal lesions was 92% in KD6 and 80% in the ST group.

Tumors from the KD8 group often showed a malignant phenotype by extending to adjacent organs (see also Supplementary Information Fig. S1). Within the other groups, a similar observation was made only in one female from the KD6 group. Although we did not find any significant differences of median tumor volume per animal between the KD4 and KD6 groups, we noticed an exponential growth of mean tumor volume per group within the ketogenic groups (R^2^ = 0.97, Fig. 2C). There were no differences between genders in all of these parameters.

### HFKD caused a drop in plasma insulin but a rise of growth hormone secretion, whereas concentrations of IGF-1 varied with the time of treatment

**Figure 3:**
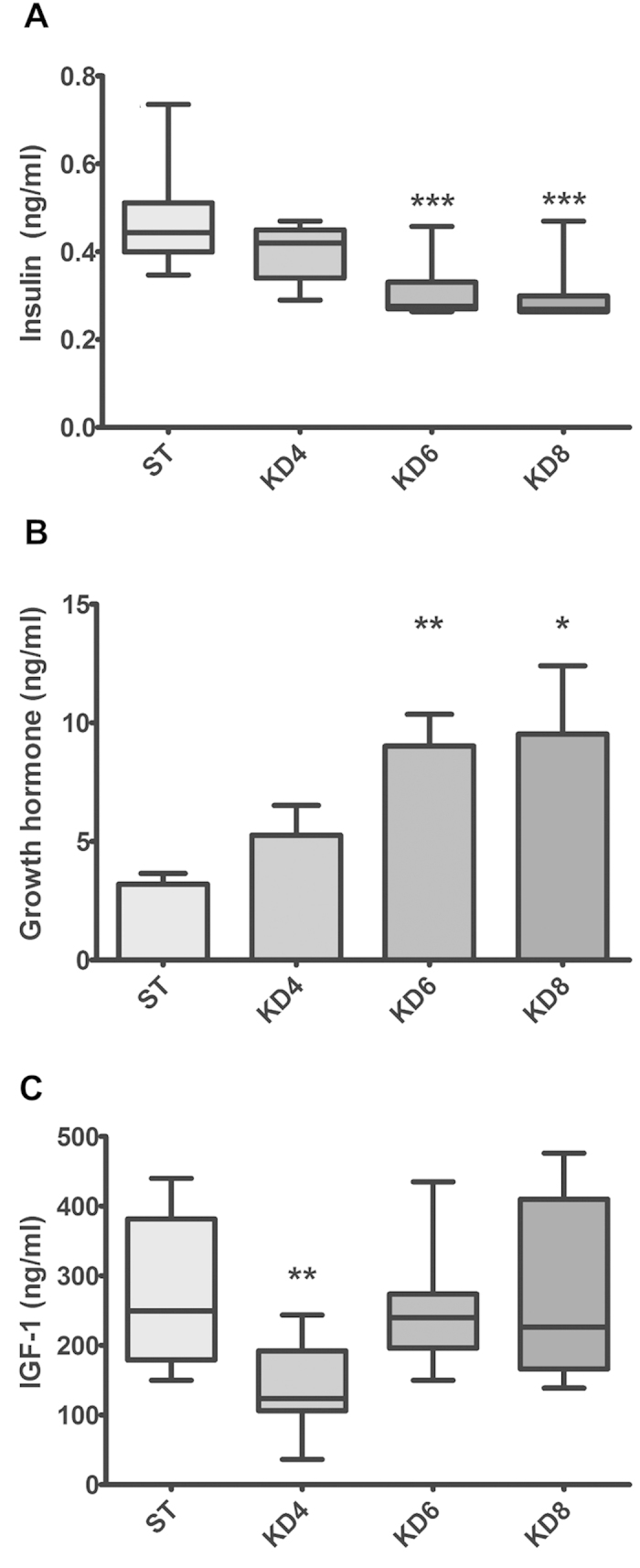
Measurements of blood protein concentrations in Eker rats (Tsc2+/−). (**A**) Insulin concentrations declined in HFKD treated rats. Growth hormone was released in ketogenic groups (**B**) whereas IGF-1 levels fluctuated depending on the time of the investigation (**C**). Results are given as a median ((**A**,**C**) whiskers represent 5–95 percentiles; Kruskal-Wallis ANOVA with a Dunn’s post hoc test) or mean ±S.E.M ((**B**) ANOVA with a Dunnett’s post hoc test). KD4, KD6, KD8 – animal groups treated with HFKD for four, six or eight mo., respectively; ST – control animals fed with a standard diet. *P < 0.05, **p < 0.01, ***p < 0.001 as compared to ST.

To gain some insight into how prolonged feeding with HFKD promotes tumor growth, we measured the plasma insulin, IGF-1 and growth hormone levels. Median plasma insulin was reduced in the KD6 (0.28 [0.3–0.5] ng/ml, p = 0.001) and KD8 (0.27 [0.3–0.5] ng/ml, p = 0.0003) but not in KD4 (0.42 [0.3–0.5] ng/ml) groups, in comparison to the control group (0.44 [0.4–0.7] ng/ml) (Fig. 3A). The insulin drop might be due to an antagonistic action of the growth hormone^27^, whose mean concentration was elevated in the rats treated with HFKD (KD4, 5.3 ± 1.3 ng/ml; KD6, 9 ± 1.3 ng/ml, p = 0.004; KD8, 9.5 ± 2.9 ng/ml, p = 0.02; Fig. 3B) as compared to control (ST, 3.2 ± 0.5 ng/ml). This was probably associated with the ability of HFKD to imitate the starvation state, in which growth hormone is the sole oversecreted anabolic hormone^28^. Furthermore, we observed changes in the concentrations of IGF-1. Initially, after four months of treatment, median IGF-1 level has declined (123 [36–243] ng/ml, p = 0.005) as compared to control (249 [150–440] ng/ml), which is in line with reports demonstrating that HFKD induces growth hormone resistance in the liver^29^. After six and eight months, the median IGF-1 concentrations returned to their normal values (240 [150–435] ng/ml in KD6 and 226 [139–476] ng/ml in KD8; p = 0.004 and p = 0.02 as compared to KD4, respectively; Fig. 3C). Such fluctuations in IGF-1 levels may be explained by the increased release of growth hormone observed in the KD6 and KD8 groups. However, an abundant release of growth hormone did not induce a proportional oversecretion of hepatic IGF-1, whose levels came back to their control values. This indicates sustained, but not total, liver resistance to the GH. Moreover, the growth hormone levels were positively correlated with tumor size within the HFKD-treated groups (r = 0.69, p < 0.001), which indicates an additional influence of HFKD on renal tumorigenesis. There were no between-sex differences in plasma levels of the examined peptides.

### HFKD activates ERK1/2 and mTOR kinases in the kidneys of Eker rats, leading to extensive growth of MAPK- and mTOR- dependent tumors

**Figure 4:**
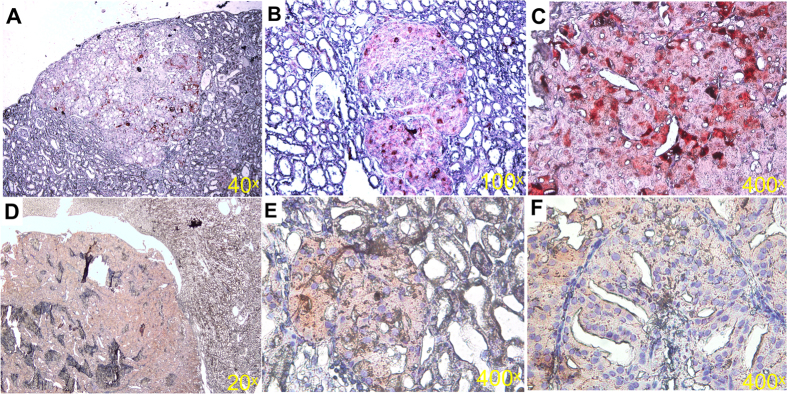
Immunohistochemistry analysis of the Eker rat (Tsc2+/−) renal tumors shows hyperactivation of ERK1/2 and mTOR kinases within lesions, relative to the surrounding tissue. Sections were prepared and stained using p-ERK1/2 Thr202/Thr204 (**A**–**C** red) and p-mTOR Ser2448 (**D**–**F** red) antibodies. Representative sections are shown.

**Figure 5:**
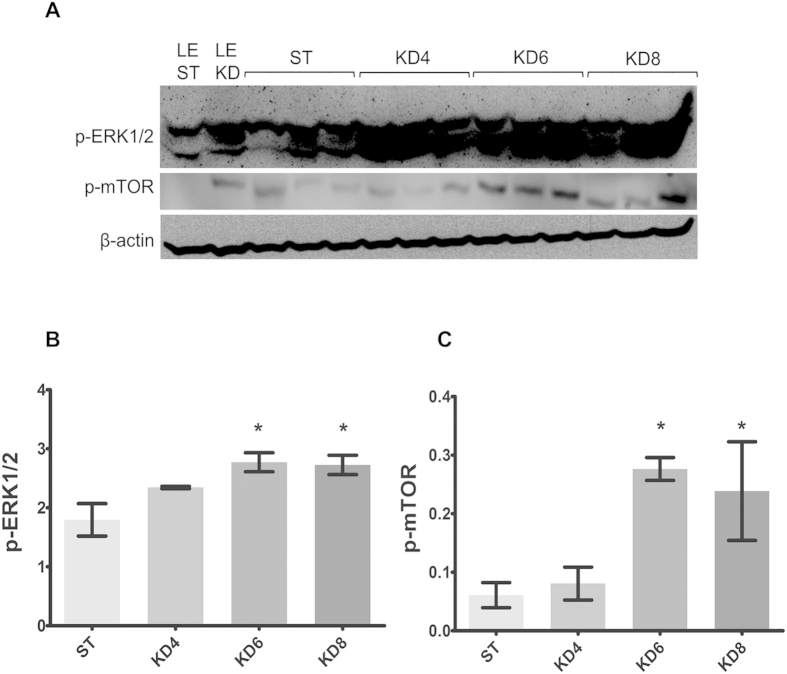
Immunoblot analysis of kidney lysates. (**A**) Blot membranes were incubated with the antibodies against p-ERK1/2 Thr 202/204 and p-mTOR Ser 2448. The mean integrated optical density is related to actin for p-ERK1/2 (**B**) and p-mTOR (**C**) activity. Results are given as a mean ± S.E.M. KD4, KD6, KD8 – groups of Eker rats (Tsc2+/−) treated with HFKD for four, six or eight mo., respectively; ST – Eker rats fed with a standard diet; LE ST – wild-type Long Evans rats treated with a standard diet; LE KD – wild-type Long Evans rats treated with a ketogenic diet similarly to the KD6 group. *P < 0.05 as compared to ST for ANOVA with a Fisher post hoc test.

It is well known that the MAPK pathway is implicated in TSC-related tumor growth^30^. Accordingly, by immunohistochemical techniques, we also observed hyperactivation of ERK1/2 in the Eker rat renal tumors (Fig. 4A–C). Moreover, we performed examinations of these proteins by Western Blotting in the renal cortex (Fig. 5A). The results showed that the MAPK pathway was highly activated in the renal tissue of HFKD-treated rats from the KD6 (p = 0.004) and KD8 (p = 0.006) groups (Fig. 5B).

Immunohistochemical labeling of phosphorylated mTOR in the kidneys revealed hyperactivation of this kinase within lesions (Fig. 4D–F). Subsequent immunoblotting analysis of renal tissue showed higher mTOR phosphorylation in the KD6 (p = 0.011) and KD8 (p = 0.027) groups (Fig. 5C).

### HFKD may prevent early tumor growth by the induction of adaptive mechanisms related to Nrf2, p53 and 8-oxoguanine glycosylase α up-regulation

**Figure 6:**
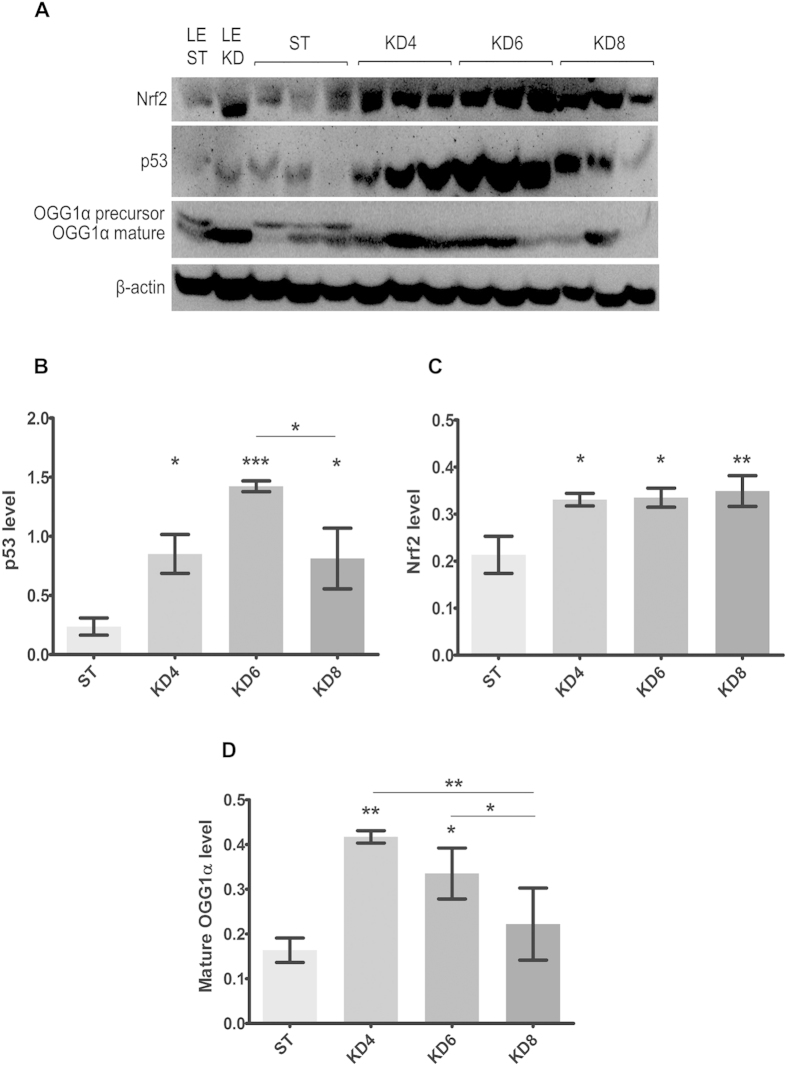
Immunoblot analysis of kidney lysates. (**A**) Blot membranes were incubated with the antibodies against Nrf2, p53 and 8-oxoguanine glycosylase α (OGG1α). The mean integrated optical density is related to actin for Nrf2 (**B**), p53 (**C**) and mature 8-oxoguanine glycosylase α (**D**) levels. Results are given as a mean ± S.E.M. KD4, KD6, KD8 – groups of Eker rats (Tsc2+/−) treated with HFKD for four, six or eight mo., respectively; ST – Eker rats fed with a standard diet; LE ST – wild-type Long Evans rats treated with a standard diet; LE KD – wild-type Long Evans rats treated with a ketogenic diet similarly to the KD6 group. *P < 0.05, **p < 0.01, ***p < 0.001 as compared to ST unless otherwise stated (horizontal line) for ANOVA with a Fisher post hoc test.

It was interesting that the pro-tumorigenic properties of HFKD were revealed only after a prolonged application of this diet. To gain insight into the mechanisms for this effect, we evaluated the possible anticancer actions of HFKD by measuring the p53, 8-oxoguanine glycosylase α and Nrf2 levels in kidney homogenates (Fig. 6A). Immunoblotting data revealed that the concentration of total p53 had been increasing with the duration of the HFKD treatment (p = 0.025 KD4 vs. ST), reaching a peak in the KD6 (p = 0.0007) group (Fig.6B). In the KD8 group, p53 levels had declined (p = 0.025 vs. KD6) but still remained higher in comparison to the control group (p = 0.032).

It is well known that ketogenic diets favor the tricarboxylic acid (TCA) cycle, where ketone bodies and fats are intensively burned leading to the generation of reactive oxygen species (ROS)^14^. It has been shown that HFKD induces ROS overproduction, which in turn stimulates up-regulation of the Nrf2 transcription factor^31^. Our analysis confirmed this data by showing that in rats fed by HFKD, the level of Nrf2 was increased (p = 0.019, p = 0.016 and p = 0.009 for KD4, KD6 and KD8 groups, respectively as compared to ST, Fig.6C).

P53 modulates 8-oxoguanine glycosylase α activity in different ways^32^. Impaired function of 8-oxoguanine glycosylase α plays a major role in renal tumorigenesis in the Eker rats^33^. An immature form of 8-oxoguanine glycosylase α (39 kDa) is cleaved to become the active protein (38 kDa) which plays a role in the conversion of oxidized guanine (8-oxoguanine) back to guanine, to avoid mutations^34^. In the ketogenic groups, only the active form of 8-oxoguanine glycosylase α was detected (Fig. 6D). Moreover, the level of mature 8-oxoguanine glycosylase α was elevated in the kidney lysates from the KD4 (p = 0.008) and KD6 (p = 0.046) groups, but not in KD8 (p = 0.008 for KD8 vs. KD4 and p = 0.028 for KD8 vs. KD6 Fig. 6D).

The antitumor properties of the HFKD, observed at the initial period of the treatment, were seen both on the molecular (Western blot analysis) and systemic (tumor size evaluation) levels. Initially, the HFKD launches adaptive molecular mechanisms marked here by upregulation of some protective proteins such as Nrf2, p53 and 8-oxoguanine glycosylase α. During this time renal tumor size did not differ significantly between HFKD (KD4 and KD6) groups and control. Tumor growth became obvious at the later period (in KD8 group) when the adaptive mechanisms were exhausted, which was accompanied by ERK and mTOR upregulation.

### HFKD increases the renal oleic acid level and affects the amino acid pool of renal tissue

**Figure 7:**
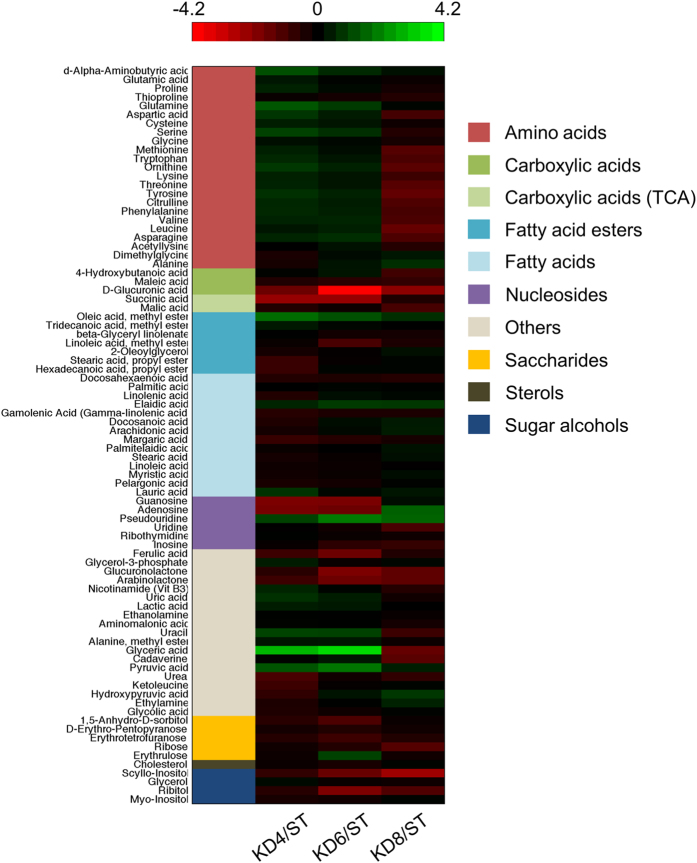
A heatmap visualisation of the changes in metabolite levels between the treatment groups KD4 (N = 4), KD6 (N = 6), KD8 (N = 5) and the control ST (N = 6). Each of the three heatmap columns represents one of the diet groups' (KD4, KD6 and KD8 respectively) log_2_ fold change in comparison to the control, with metabolite levels averaged across all of the specimens within each group. The compound group that each metabolite belongs to is colour-coded in the first column of the heatmap, with the mapping provided in the legend. Black indicates a log_2_ fold change of zero, representing a lack of change between the treatment group and the control. Increasing brightness of green indicates a higher level of the metabolite in the treatment group in comparison to the control (log_2_ fold change >0). Increasing brightness of red indicates a lower level of the metabolite in the treatment group in comparison to the control (log_2_ fold change <0). The log_2_ fold change corresponding to a given colour on the heatmap can be elucidated from the colour bar.

In order to reveal the pro-tumorigenic effects of other variables arising from the prolonged HFKD treatment, GC/MS-based profiling of the metabolites from the rats’ kidney tissue extracts was performed. This approach allowed for the identification of many metabolites most of which were assigned to the following clusters: amino acids, carboxylic acids, fatty acids esters, fatty acids, nucleosides, saccharides, sterols, sugar alcohols and others. The obtained data revealed that using the HFKD for 4, 6 and 8 mo. altered metabolome composition (Fig. 7). However, significant differences were noticed only for some metabolites (Table 1). We observed an increased accumulation of oleic acid in rats treated with HFKD. It should be also emphasized that 5 other metabolites: guanosine, D-glucuronic acid, glyceric acid, scyllo-inositol and succinic acid significantly differed between the groups (Table 1). We have also performed an additional analysis between all metabolite clusters by comparing relative levels (mean value for each metabolite related to the corresponding mean value in the control group) of all metabolites within a cluster between KD4, KD6 and KD8. As a result, we have observed a statistical difference only between amino acids clusters. The amino acid pool was significantly lower in KD8 as compared to KD4 and KD6 (p < 0.001 for both, ANOVA with Tukey post hoc test, Fig. 7).

## Discussion

HFKD is a well appreciated anti-seizure therapy for TSC, but it has not been clearly established how it may affect the tumor growth related to this disorder. In a case series the HFKD was not sufficiently protective against progression of tumors and was not seen to induce tumor regression in TSC^35^. Herein, we have shown for the first time that HFKD promotes renal tumor growth in a rat model of TSC. We designed our experiment to reveal the long-term effects of unrestricted HFKD on tumorigenesis and to discriminate its pro-tumorigenic properties. In contrast to our results, some previous investigations conducted on animal models with tumor xenografts showed that HFKD suppressed the development of tumors. HFKD, with caloric restrictions, has been shown to reduce glioma development in mice^36^. However, its administration in an ad libitum manner did not reveal these antitumor properties^37^,^38^,^39^. Similarly, the Freedland team released a series of reports showing that long-term treatment with unrestricted HFKD (for 2–3 months) did not affect the xenograft prostate cancer growth in mice^40^,^41^,^42^. Therefore, it is probable that the observed antitumor effect depended mostly on the calorie restriction associated with HFKD, but not on the diet itself, especially given that such antitumor properties of calorie restriction have been well documented in other animal cancer models^43^.

However, some reports from animal models with injected cancer cells have also shown that unrestricted HFKD delayed tumor growth^44^,^45^,^46^,^47^. These investigations were performed on specialized cancer cell lines which had acquired many mutations, and it is probable that in these cells, the metabolism strongly depended on the Warburg effect. Poff *et al*. showed that HFKD suppressed the growth of CT-2A cells^44^ which had impaired mitochondrial metabolism and depended mainly on glycolysis^48^. Furthermore, Otto *et al*. showed a decrease of 23132/87 adenocarcinoma in mice subjected to unrestricted HFKD^45^. This cell line carries many mutations, among others c-myc hyperactivity^49^, which stimulates aerobic glycolysis^50^. Similarly, Stafford *et al*.^46^ and Abdelwahab *et al*.^47^ investigated GL-261 cells which also exhibited c-myc hyperactivity^51^. So, in all of these cases of glycolysis-dependent tumors, the carbohydrate restriction that occurs under HFKD may be responsible for the observed inhibition of growth.

Considering all of the above, it is possible that the antitumor effects of HFKD depend mainly on the accompanying calorie restrictions, and in the case of glycolysis-dependent neoplasms, the limitation of carbohydrates is sufficient for the inhibition of tumor growth. Such an assumption is confirmed by the results of this study, where the size of naturally occurring tumors did not differ between the animals fed with a standard fodder or with unrestricted HFKD for the periods of 4 and 6 months. However, after 8 mo. of HFKD feeding, we observed an increased development of renal tumors. Additionally, in the abovementioned studies, HFKD was used in the animals for no longer than 3 mo. Taking into account our results, the prolonged feeding with HFKD may not only have no antitumor properties, but can even lead to tumor growth.

We have also shed some light on the mechanisms launched by HFKD, which may be implicated in tumor physiology. We hypothesized that the up-regulation of ERK1/2 could be connected with an increased supply of triglycerides in the ketogenic groups. We didn’t notice an elevation of blood triglycerides in the HFKD-treated animals, but we observed an accumulation of oleic acid in the kidneys. There was a significant increase in the renal oleic acid levels within all the ketogenic groups. It has been shown that oleic acid induces ERK1/2 activation^52^ leading to cancer progression, and can stimulate the growth of renal carcinoma cells^53^, which supports our hypothesis about the pro-tumorigenic effects of HFKD. Likewise, it has been shown that the growth hormone activates the MAPK pathway, thus its overproduction in the ketogenic groups may also boost ERK1/2 phosphorylation^54^,^55^. We believe that HFKD induced the ERK1/2 activation results as a cumulative effect of the renal oleic acid accumulation and the systemic growth hormone overproduction. Additionally, we observed hyperactivation of mTOR kinase in the HFKD treated groups, which also may stimulate growth of mTOR dependent tumors in Eker rats. It is possible that mTOR was stimulated by ERK1/2^56^.

Although the amount of most metabolites fluctuated between the groups, only some substances exhibited significant changes. Unfortunately, in line with current knowledge, we are not able to clearly explain such fluctuations. In particular, the decline in the levels of succinic acid in the KD4 and KD6 groups should be discussed because of its implication in cancerogenesis^57^. Its drop within these groups may be a result of an intensive TCA cycle turnover, as a consequence of high ketone bodies utilization. However, succinate fluctuations may be also related to other processes, because another TCA intermediate – malic acid – has not exhibited a quantitative difference.

Furthermore, the comparison of metabolite clusters revealed a lower level of amino acids in the KD8 group. It is well known that the progression of cancer is accompanied by impaired protein metabolism, with one of the causes being an increased utilization of amino acids to form glucose^58^. This process may be particularly enhanced under prolonged HFKD-induced carbohydrate depletion. Additionally, amino acids are susceptible to oxidation, resulting in the degradation to distinct products^59^. The amino acid levels in KD8 might also decrease due to the chronic elevation of oxidative stress, as a consequence of prolonged treatment with HFKD. It appears that the change of the amino acid pool observed in KD8, when compared to the KD4 and KD6 groups, corresponds well to the time dependent protumorigenic properties of HFKD.

In parallel, we observed that HFKD stimulates protective mechanisms against cancer by the stimulation of p53, Nfr2 and 8-oxoguanine glycosylase α. P53 functions as a tumor suppressor through regulating the cell cycle and promoting apoptosis in order to prevent the accumulation of genome mutations. Additionally, p53 and 8-oxoguanine glycosylase α proteins participate in DNA repairing, and a higher level of these proteins prevents normal cells from the expression of a mutator phenotype^60^. 8-oxoguanine glycosylase α is activated in response to oxidative stress; thus, HFKD-induced ROS overproduction (marked by Nrf2) can explain the higher levels of the 8-oxoguanine glycosylase α mature form in the ketogenic groups. This process may be enhanced by interaction with p53^32^. Taking all of the above into consideration, it seems that HFKD-induced stress stimulates DNA-reparative mechanisms, which are responsible for the anti-cancer properties of this diet. Lower levels of p53 and 8-oxoguanine glycosylase α in the KD8 group, in comparison to the shorter times of HFKD treatment, may be the reason for the loss of the anti-tumorigenic properties of HFKD under prolonged treatment.

Our study shows that long-term feeding by HFKD results in a higher incidence, as well as a bigger size, of renal tumors in Eker rats. HFKD simultaneously launches opposite mechanisms: pro-tumorigenic and anti-cancerous. The first one is progressive during the time of the HFKD application, and depends on up-regulation of intracellular anabolic pathways. This leads to the development of mTOR and MAPK-dependent tumors found in the kidneys of Eker rats. The second is an anti-cancerous mechanism associated with HFKD – induced intracellular stress and leads to an adaptive response which may provide protection against neoplastic transformations. The anti-cancerous mechanism is protective at some initial period, but it fails with the increased duration of HFKD usage, uncovering the pro-tumorigenic hallmarks of this diet.

## Additional Information

**How to cite this article**: Liśkiewicz, A. D. *et al*. Long-term High Fat Ketogenic Diet Promotes Renal Tumor Growth in a Rat Model of Tuberous Sclerosis. *Sci. Rep.* **6**, 21807; doi: 10.1038/srep21807 (2016).

## Supplementary Material

Supplementary Information

## Figures and Tables

**Table 1:** Metabolomic profiling of kidneys in Eker rats (Tsc2+/−) from ST (N = 6), KD4 (N = 4), KD6 (N = 6) and KD8 (N = 5) groups.

	ST	KD4	KD6	KD8
D-Glucuronic acid	150 ± 34	38 ± 29	3.8 ± 1.5**	28 ± 26*
Glyceric acid	0.34 ± 0.09	2.74 ± 0.65**	4.12 ± 1.28**	0.11 ± 0.02
Guanosine	0.7 ± 0.19	0.17±0.06*	0.17 ± 0.07**	0.8 ± 0.23
Oleic acid	0.16 ± 0.01	0.57 ± 0.12**	0.41 ± 0.04**	0.28 ± 0.03*
Scyllo-Inositol	3.6 ± 0.53	2.1 ± 0.45	1.1 ± 0.26*	0.46 ± 0.13**
Succinic acid	1.37 ± 0.46	0.24 ± 0.04**	0.25 ± 0.06**	0.96 ± 0.24

Values represent mean fold change ± SEM. *P < 0.05, **p < 0.01 respectively to the ST group (ANOVA with a Tukey’s post hoc test). KD4, KD6, KD8 – groups treated with HFKD for four, six or eight mo., respectively; ST – control animals fed with a standard diet.
