# Supplementary material for: Long-term High Fat Ketogenic Diet Promotes Renal Tumor Growth in a Rat Model of Tuberous Sclerosis
Source: Sci Rep. 2016 Feb 19;6:21807. doi: 10.1038/srep21807 (PMC4759602; doi:10.1038/srep21807)
Supplement: Supplementary Information [file srep21807-s1.pdf]

Supplementary information

Long-term High Fat Ketogenic Diet Promotes Renal Tumor Growth in a Rat Model of Tuberous Sclerosis

Arkadiusz D. Liśkiewicz [1, 2 *], Daniela Kasprowska [3], Anna Wojakowska [4], Krzysztof Polański [5], Joanna Lewin – Kowalik [1], Katarzyna Kotulska [6], Halina Jędrzejowska – Szypułka[1]

[1] Medical University of Silesia, School of Medicine in Katowice, Department of Physiology, Katowice, Poland

[2] Affective Cognitive Neuroscience Lab, Department of Behavioral Neuroscience and Drug Development, Institute of Pharmacology Polish Academy of Sciences Krakow, Poland

[3] Medical University of Silesia, School of Medicine in Katowice, Department of Pharmacology, Katowice, Poland

[4] Maria Sklodowska-Curie Memorial Cancer Center and Institute of Oncology, Gliwice Branch, Poland.

[5] Warwick Systems Biology Centre, University of Warwick, CV4 7AL, UK.

[6] Department of Neurology and Epileptology, The Children's Memorial Health Institute, Warsaw, Poland

*Corresponding author:

Medyków 18

40-752 Katowice, Poland

E-mail: adliskiewicz@gmail.com

Telephone number: +48 32 252 50 87

Supplementary data

Figure S1 Representative photographs of the renal tumors identified in the Eker rats. A-F – the tumors observed in KD8 group (animal treated with HFKD for eight mo.), photographed on bold one-centimeter grid lines. G,H – typical renal tumor in standard diet fed rats (ST group).

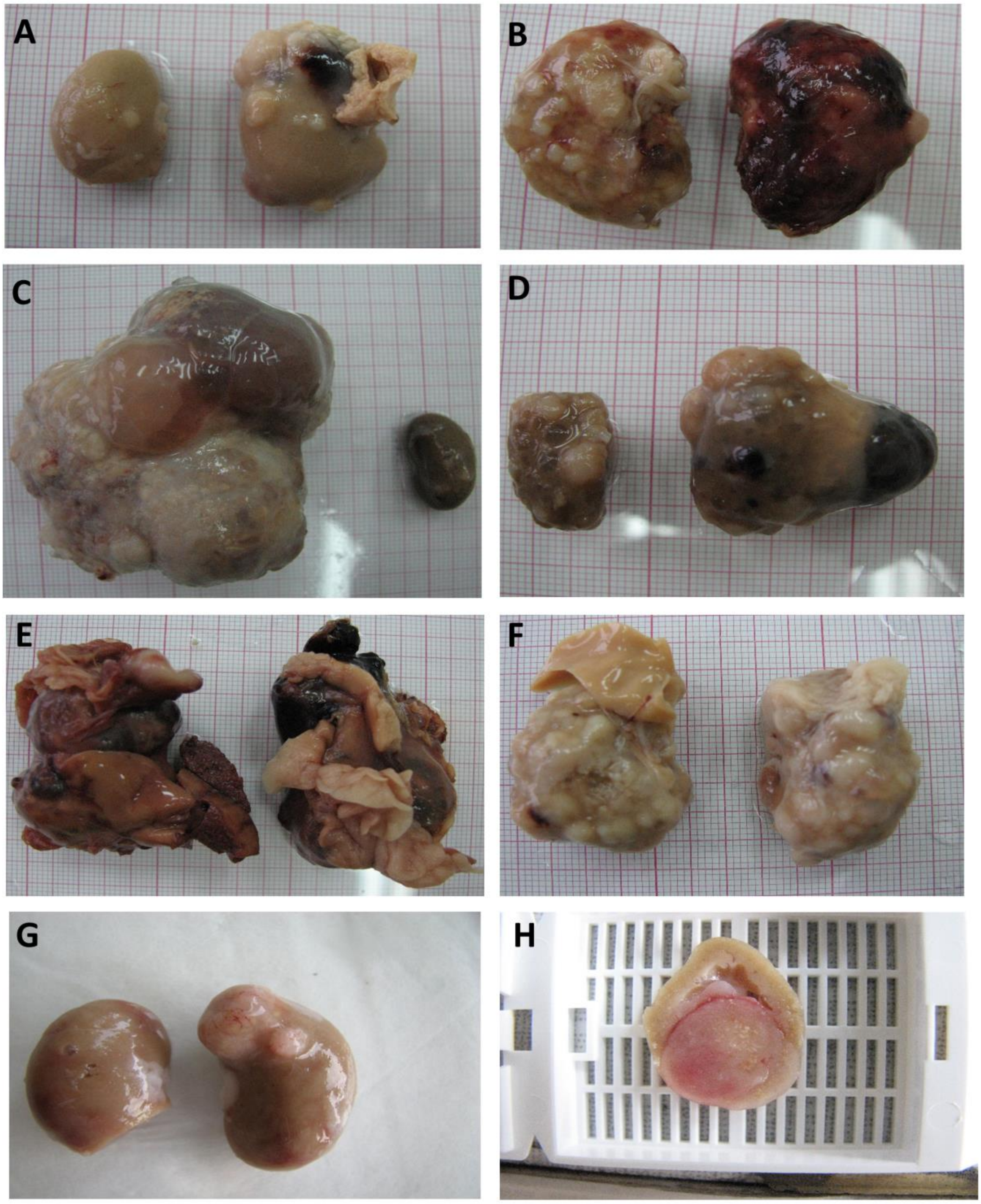

Supplementary Materials and Methods

Composition of a high fat low carbohydrate ketogenic diet (HFKD) (Supplementary Table S1, S2, S3, S4).

Supplementary Table S1 The composition of the raw materials used in HFKD preparation.

Component	HFKD (%)
Lard	47,809
Butter	20
Corn oil	11
Casein	9,5
Dextrose	0,75
Wheat bran	5
Vitamin and mineral mix	5,941
Total	100

Supplementary Table S2 Vitamin composition of HFKD and standard rodent diet.

Vitamin content in 1 kg of fodder		
**Component**	**Standard rodent diet**	**High fat ketogenic diet**
Vitamin A (IU)	12 000	15 000
Vitamin D3 (IU)	800	1 000
Vitamin E (mg)	80	91
Vitamin K3 (mg)	2,4	3
Vitamin B1 (mg)	18	18,04
Vitamin B2 (mg)	12	15,2
Vitamin B6 (mg)	15	13,6
Vitamin B12 (µg)	60	40,1
Vitamin (mg)	25	34,5
Folic acid (mg)	4	4,26
Nicotinic acid (mg)	100	75,1
Biotin (mg)	0,3	0,254

Supplementary Table S3 Vitamin composition of HFKD and standard rodent diet.

Mineral content in 1 kg of fodder		
**Component**	**Standard rodent diet**	**High fat ketogenic diet**
Calcium (g)	10,0	10,2
Phosphorus (g)	7,5	6,51
Magnesium (g)	3	2,97
Sodium (g)	2,2	1,1
Iron (mg)	250	200,1
Manganese (mg)	100	100,4
Zinc (mg)	100	104
Copper (mg)	20	15,38
Iodine (mg)	0,8	0,15
Selenium (mg)	0,4	0,4

Supplementary Table S4 Amino acid composition of HFKD and standard rodent diet.

Amino acid content in 1 kg od fodder		
**Component**	**Standard rodent diet**	**High fat ketogenic diet**
Lysine (g)	10	6,42
Methionine (g)	3,2	2,6
Tryptophan (g)	2	1,01
Threonin (g)	7	3,49
Isoleucine (g)	5,6	4,87
Leucine (g)	12	7,97
Valine (g)	8	5,8
Histidine (g)	4	2,4
Arginine (g)	9	3,1
Phenyloalanine (g)	7	4
Tyrosine (g)	5,5	4,83

Western Blotting

The frozen renal tissues (cortex) were homogenized by Tissue Ruptor (QIAGEN, Valencia, CA, USA) with RIPA lysis buffer (Millipore, St. Charles, MO, USA) supplemented with Complete Protease inhibitors and PhosphoSTOP phosphatase inhibitors (both from Roche, Indianapolis, IN, USA). After 15 min on ice, the tissue lysates were centrifuged at 15,000 g for 15 min at 4°C. The protein level of tissue extracts was determined by using Rotiquant Protein Assay (Carl Roth GmbH, Karlsruhe, Germany) with serum albumin as a standard. Samples containing equal amounts of protein (20-50 µg) were boiled in LI-COR Protein Loading Buffer (Licor Inc. Lincoln, Nebraska, USA) for 3 min, separated on 10% SDS-polyacrylamide gels

and transferred to polyvinylidene difluoride (PVDF) membranes. Non-specific binding was inhibited by incubation in TBST (20 mM Tris-buffered saline [pH 7.5] with 0.1% Tween 20) containing 5% non-fat dried milk for 1 h at room temperature (RT).

The membranes were then incubated overnight at 4°C with primary antibodies diluted in TBST containing 5% (w/v) non-fat milk, then washed with TBST and incubated at RT for 60 min with an appropriate HRP-conjugated secondary antibody diluted to 1:1000 or 1:2000, and then washed three times, with each wash taking 10 min, with TBST. The blots were then developed using the chemiluminescence ECL kit and detected digitally with a ChemiDoc-IT 410 system (UVP, Upland, CA, USA). The molecular weights of examined proteins were estimated through the electrophoresis of prestained protein markers (ColorPlus™ Prestained Protein Ladder, Broad Range [10-230 kDa], New England Biolabs Inc., Ipswich, MA, USA). To evaluate the results of the Western blot analysis, each band was quantified by densitometry using the ImageJ (NIH, Bethesda, MD, USA) analysis software.

Metabolome analysis

Extraction of metabolites. 10 mg of rat renal cortex were homogenized in 250 µl of the mixture of MeOH:H_2O (1:1 v/v), mixed using a vortex mixer for 5 min and placed in an ultrasonic bath for 10 min. The resulting mixture was centrifuged for 10 min at 16 000 rpm in 4°C. The supernatant was transferred to centrifugal filter units (Millipore PVDF 0,22 µm), and then centrifuged for 1min at 13000 rpm to generate polar fraction. The pellet was suspended in 250 µl of mixture of Ch_2Cl_2:MeOH (3:1 v/v) and processed as described above to generate non-polar fraction. Both polar and non-polar fractions were combined, transferred to a new tube and evaporated in a SpeedVac concentrator. Hexadecanoic acid-1-^{13}C (10 µl of methanol solution at a concentration of 1 mg/ml) was added to each sample as an internal standard. The dried extract was then derivatized with 25 µl of methoxyamine hydrochloride in pyridine (20 mg/ml) at 37°C for 90 min with agitation. The second step of derivatization was performed by adding 40 µl of MSTFA and incubation at 37°C for 30 min with agitation. The samples were subjected to GC/MS analysis directly after derivatization. Compounds were treated as identified when they met the identification criteria established by GC software used (LECO ChromaTOF), namely they were:

identity score higher than 700, suitable intensity threshold and matching retention index.

GC/MS analysis. The analysis was performed using Agilent 7890A gas chromatograph (Agilent Technologies) connected to Pegasus 4D GCxGC-TOFMS mass spectrometer (Leco). A DB-5 bonded-phase fused-silica capillary column of 30 m length, 0.25 mm inner diameter and 0.25 μm film thickness (J&W Scientific Co., USA) was used for separation. The GC oven temperature program was as follows: 2 min at 70°C, raised by 8°C/min to 300°C and held for 16 min at 300°C. The total time of GC analysis was 46.75 min. Helium was used as the carrier gas at a flow rate of 1 mL/min. One microliter of each derivatized sample was injected in splitless mode. The initial injector temperature was 20°C for 0.1 min and after that time raised by 600°C/min to 350°C. The septum purge flow rate was 3 mL/min and the purge was turned on after 60 s. The transfer line and ion source temperatures were set to 250°C. In-source fragmentation was performed by applying 70 eV. Mass spectra were recorded in the mass range of 35-650 m/z.

Analysis of spectra. Data acquisition, automatic peak detection, mass spectrum deconvolution, retention index calculation and library search were performed with Leco ChromaTOF-GC software (v4.51.6.0). To eliminate retention time (Rt) shift and to determine the retention indexes (RI) for each compound, the alkane series mixture (C-10 to C-36) was injected into the GC/MS system. The metabolites were automatically identified through a library search (Replib, Mainlib, Fiehn library) with a similarity index threshold (SI) above 700 and RI +/-10. All known artefact peaks including alkanes, plasticizers, column bleed, MSTFA artefact and reagent peaks were not taken under consideration in the final results. To obtain accurate peak areas for the deconvoluted components, unique quantification masses for each component were specified and the samples were reprocessed. The obtained metabolite data were normalized relative to the internal standard and sample mass before statistical analyses.

Statistical analysis of metobolomic data

In the metabolomic profiling, the data was filtered to only feature metabolites that showed up in 100% of the samples in each of the groups. The data was log-

transformed, the normality was assessed with the Lilliefors composite goodness-of-fit test for each group, and homoscedasticity was assessed with Bartlett's test. The data for each individual metabolite was analyzed with both ANOVA and a Kruskal-Wallis one-way analysis of variance. The P-values for each group's normality testing, homoscedasticity testing, ANOVA and Kruskal-Wallis testing had the Benjamini-Hochberg FDR correction applied. After the FDR correction, the final test was chosen after assessing the normality and homoscedasticity of each metabolite's data across the groups: if at least one of the groups was identified as not normal, or the Bartlett's test P-value was significant, the Kruskal-Wallis was chosen as the test for the metabolite; otherwise, ANOVA was used as the test for the metabolite. If the final chosen test P-value was significant (with a significance threshold of 0.05), post-hoc testing was also performed to identify between which groups the values were significantly different. In the case of ANOVA Tukey test was used. In the case of Kruskal-Wallis, the Nemenyi test with the Dunn correction was used. The test statistic was compared to the tabulated values for a significance threshold of 0.05.
